# CULTURAL RESOURCES AND PSYCHOLOGICAL WELL-BEING INFLUENCES ON COGNITIVE FUNCTION IN MEXICAN AMERICAN IMMIGRANTS

**DOI:** 10.1093/geroni/igae098.1155

**Published:** 2024-12-31

**Authors:** Jeung Hyun Kim, Merril Silverstein

**Affiliations:** Syracuse University, Syracuse, New York, United States; Syracuse University, Syracuse, New York, United States

## Abstract

The present research investigates the mechanisms through which different cultural resources retained by the very old Mexican American immigrants shape their psychological and cognitive wellbeing. The very old immigrants are likely to adhere to the traditional values originated from their country of origin and may be isolated from the mainstream culture. The roles that acculturation and the availability of ethnocultural resources like familismo and religious practices play can generate their loneliness and depression, which then, lead to their cognitive health disparities. It uses HEPESE (Wave 7), the epidemiological study of older Mexican Americans residing in the Southwestern U.S. Path models are utilized for the analysis. Of 368 very old immigrants, results reveal that stronger intergenerational ties and marriage reduce loneliness while increased acculturation, immigrating at a younger age as a proxy measure of acculturation, and fewer religious engagement elevate depression. Though there is a marginal association between the psychological wellbeing and the global cognitive health, cognitively impaired individuals are likely to associate with their adult children more. When stratified across cognitive dimensions, immigrating at an older age and depression demise their memory function. A more active religious engagement lessens their depression, which can further strengthen their memory function. Regarding the nonmemory function of the immigrants, their cultural resources and psychological wellbeing do not show significance. The discussion focuses on how the possession of ethnocultural resources renders benefits to the very old immigrants across health dimensions while their acculturation, age-at-immigration, has different influences on their psychological and cognitive wellbeing.

